# Racial Differences in Length of Stay for Patients Who Leave Against Medical Advice from U.S. General Hospitals

**DOI:** 10.3390/ijerph13010095

**Published:** 2015-12-31

**Authors:** Rima Tawk, Matthew Dutton

**Affiliations:** Institute of Public Health, College of Pharmacy and Pharmaceutical Sciences, Florida A&M University, Tallahassee, FL 32307, USA; matthew.dutton@famu.edu

**Keywords:** against medical advice, treatment refusal, length of stay, health disparities, social determinants of health.

## Abstract

There is a paucity of published literature on the length of hospital stays (LOS) for patients who leave against medical advice (AMA) and on the factors that predict their LOS. The purpose of the study is to examine the relationship between race and the LOS for AMA patients after adjusting for patient and hospital characteristics. National Hospital Discharge Survey (NHDS) data were used to describe LOS for AMA patients aged 18 years or older. Patient characteristics included age, sex, race, marital status, insurance, and diagnosis (ICD-9-CM). Hospital characteristics consisted of ownership, region and bed size. LOS was the major outcome measure. Using data from all years 1988–2006, the expected time to AMA discharge was first examined as a function of race, then adjusting for year terms, patient and hospital characteristics, and major medical diagnoses and mental illness. The unadjusted effect of race on the expected time of leaving AMA was about twice the adjusted effect. After controlling for the other covariates, the expected time to AMA discharge is 20% shorter for Blacks than Whites. The most significant predictors included age, insurance coverage, mental illness, gender, and region. Factors identified in this study offer insights into directions for evidence based- health policy to reduce AMA discharges.

## 1. Introduction

Against Medical Advice (AMA) discharge occurs when a patient decides to leave the hospital before the physician has recommended discharge. A review of the literature revealed numerous articles over the last four decades concerned with rates, patient characteristics, and predictive factors. An obvious feature is the great variation seen depending upon medical conditions, patient populations, and type of treatment settings [1,2]. Younger age, male gender, insurance status, personality disorders, and substance abuse have been the most consistent characteristics shared by patients discharged AMA from general hospitals [3,4]. In the previous studies that examined race, most [3,4,5,6,7,8,9] but not all [10,11], studies have found that African American patients have an increased risk of AMA discharges compared with white patients. The literature suggests that the relationship between race and AMA discharge could reflect the known association between lower socioeconomic status and AMA discharge [3,8,11,12]. More recent studies however found no relationship between race/ethnicity and AMA discharge after adjusting for individual and hospital socioeconomic factors such as metropolitan size, percent medicaid, or percent minorities [2,13]. Others suggested that racial discrimination and poor communication at the individual level are not primary factors in AMA discharges, but that place of hospitalization, income, and insurance, collectively referred to as ‘‘structural racism’’ do contribute to AMA discharges [13].

The medical literature supports the view that patients who leave AMA have worse outcomes than patients without such discharges and are at risk for early readmission for selected conditions such as alcohol abuse, asthma, and HIV-related complications [7,8,14]. Previous studies found out that hospitalizations that end AMA are three times more likely to result in readmission within 30 days [2,14]. Thirty-four percent of these readmissions were to a higher level of care because of worsened clinical presentations resulting from significantly shorter lengths of stay and incomplete treatment [8,11,15,16,17,18,19]. 

AMA discharge remains a significant health care problem since it places the patients at risk for adverse medical outcomes including mortality [20,21], disrupts the patient-provider relationship, and results in higher, unnecessary health care costs for the subsequent care of an initially inadequately treated condition [5,6,17,20,21,22,23,24]. For conditions such as inadequately treated meningitis, endocarditis, diabetic ketoacidosis, or even pneumonia, failure to complete treatment can be overwhelming. Follow-up care may be more difficult and more costly [9]. 

Research examining race and ethnicity has found longer LOS for White *versus* African American or Hispanic patients [25]. There is a paucity of published literature on the length of stay for patients who leave AMA and on the factors that predict their length of stay (LOS). The purpose of the study is to determine which patient and hospital characteristics are significantly associated with the LOS for the major racial groups of U.S. AMA populations. The hypotheses for this study were as follows: (1) there is an association between race and the length of stay among AMA patients; (2) patient, clinical, and hospital characteristics explained partly the relationship between the length of stay and race for AMA patients. 

## 2. Methods

The study is a secondary data analysis from the National Hospital Discharge Survey (NHDS) conducted by the National Center for Health Statistics (NCHS) which uses the official version of International Classification of Diseases, 9th revision Clinical modification (ICD 9-CM). NHDS, which has been conducted annually since 1965, provides national estimates of hospitalizations in nonfederal short stay hospitals. NHDS, described in detail elsewhere [26] annually collects data from a sample of approximately 300,000 inpatient medical records from a national sample of about 500 nonfederal short-stay care hospitals. The survey used a stratified multistage cluster sampling design. The NHDS is based on a three-stage sampling design. The first stage selects a probability sample of primary sampling units. The second stage selects all hospitals within the chosen primary sampling units that have 1000 or more beds or 40,000 or more annual discharges and a probability sample of smaller hospitals. The third stage selects discharges from the chosen hospitals by systematic random sampling. This strategy oversamples frequent users of inpatient care but accurately measures overall provision of inpatient care in the sampling frame. 

The NHDS provides national estimates of characteristics of patients, length of stays, diagnoses, surgical and non-surgical procedures in general hospitals of various bed sizes, ownerships, and geographic regions of the U.S.. The information provides a “snapshot” of the conditions for which Americans are hospitalized each year. The NHDS provides a record for each hospital discharge episode, not for each individual patient. The unit of analysis is the hospital discharge. NHDS data were used to describe the utilization patterns for AMA adult patients. Patient characteristics included age, sex, race, marital status, insurance, and diagnosis (ICD-9-CM). Race was categorized as Whites, Blacks, and other race. Other race included American Indian/Alaskan native, Asian, native Hawaiian/other Pacific Islander, other, and not stated. Marital status was coded as married, single, and other. Other included widow, divorced, separated, and not stated. The expected primary source of payment was categorized as private insurance, medicaid, medicare, self-pay, and other. Private insurance included Blue Cross/Blue Shield, health maintenance organizations (HMO)/or preferred provider organizations (PPO), and other private insurance. Other insurance included worker’s compensation, other government payments, no charge, and other insurance. NHDS dataset includes up to seven diagnoses per discharge. Major medical illnesses include circulatory, digestive, respiratory, poisoning, endocrine, and infectious diseases. Mental illness in NHDS data includes alcohol abuse, drug abuse, psychosis, and mood disorder. Hospital characteristics consisted of ownership, region and bed size. Hospital ownership refers to not for profit, for profit, and government. Geographical location refers to regional distribution of hospitals and was coded according to the U.S. Census Bureau's classification as Northeast, Midwest, South, or West. The months of discharge were coded into four seasons: winter, spring, summer, and fall to determine whether there is a seasonal variation in AMA discharge. Among patients discharged alive, hospitals reported discharge disposition as routine discharge/discharged home, left against medical advice, transferred to another hospital, transferred to a long-term care institution, or other disposition. NHDS relies on the accuracy of what health care professionals record in a patient’s medical record. Hospital records are unlikely to be incorrect on whether a patient was discharged against medical advice. Patients aged less than 18 were excluded. Discharges resulting in patient death or discharges with missing patient age or diagnosis were excluded. Discharges with length of stay greater than 30 days were also excluded from the analysis. 

### Ethics Statement

The proposal for this study was reviewed and approved by the Institutional Review Board of the University of Illinois at Chicago as an exempt study (protocol # 2007-0230). 

## 3. Materials and Statistical Analysis

LOS was the major outcome measure by using cause specific survival model. LOS was defined as the number of days between the date of admission to the date of AMA discharge. Patients who were discharged home, or transferred to short-term facility or long-term care institution or alive with disposition not stated at end of study or unstated discharge status during study period were statistically censored. Statistical analyses were performed using parametric survival regression (PROC LIFEREG) in SAS Version 9.4 (SAS Institute Inc., Cary, NC, USA). Using data from all years 1988–2006, the expected time to AMA discharge was first examined as a function of race, then adjusting for year terms, patient and hospital characteristics, and major medical diagnoses and mental illness. 

## 4. Results

We used a sample of 45,948 hospital AMA discharge records representing a weighted total of 4,950,431. The number of observations that were censored consisted of 4,391,638. Descriptive statistics were used to summarize patient characteristics (Table 1 and Table 2). Table 2 displays the demographic characteristics of the patients in each of the three groups. AMA patients included a greater proportion of whites (58.0%) while Blacks constituted 20.4% of discharges and other race 21.6%. Males constituted the majority of discharges (59.2%). The highest frequency (57%) of signing out AMA pertained to the 18–44 years old group. About 27% were single while 54.7% were either widowed, divorced, or separated or unknown marital status. Study population included a greater proportion of patients on Medicaid (26.1%). Seventy-two percent of AMA discharges occurred in not for profit hospitals. A greater proportion of AMA discharges occurred in the Northeast. Most of AMA discharges occurred in medium size hospitals (100–199) 26.9%. Majority of AMA discharges occurred in the spring. About 70% of AMA discharges occurred within 3 days of arrival. Significant differences were found in every category examined except for season.

**Table 1:** AMA hospital stay characteristics by racial categories.

Racial Categories	Unweighted *N* 45,948	Weighted *N* 4,950,431	Weighted %
White	24,457	2,869,055	58.0
Black	10,895	1,010,251	20.4
Other	10,596	1,071,125	21.6

**Table 2:** Characteristics of hospital stays for AMA by racial groups.

Variable	Categories	White		Black		Other		*p* Value
		Weighted *N* 2,869,055	Weighted (%) *	Weighted *N* 1,010,251	Weighted (%)	Weighted *N* 1,071,125	Weighted (%)	
Age	18–44	1,531,725	31.0	661,933	13.4	634,170	12.8	<0.0001
	45 to 64	791,912	16.0	264,467	5.3	282,790	5.7	
	65+	545,418	11.0	83,851	1.7	154,165	3.1	
Gender	Male	1,676,058	33.9	603,060	12.2	648,010	13.1	0.0700
	Female	1,192,997	24.1	407,191	8.2	423,115	8.5	
Marital Status	Married	676,798	13.7	106,082	2.1	144,377	2.9	<0.0001
	Single	693,064	14.0	414,579	8.4	209,041	4.2	
	Other	1,499,193	30.3	489,590	9.9	717,707	14.5	
Insurance	Private	687,717	13.9	147,765	3.0	217,251	4.4	<0.0001
	Medicaid	576,190	11.6	394,616	8.0	323,209	6.5	
	Medicare	802,167	16.2	191,762	3.9	238,391	4.8	
	Selfpay	460,954	9.3	160,966	3.3	195,368	3.9	
	other	342,027	6.9	115,142	2.3	96,906	2.0	
Bedsize	6–99	633,963	12.8	85,098	1.7	221,357	4.5	<0.0001
	100–199	858,288	17.3	182,907	3.7	290,022	5.9	
	200–299	480,409	9.7	227,056	4.6	181,151	3.7	
	300–499	640,273	12.9	312,393	6.3	269,500	5.4	
	500+	256,122	5.2	202,797	4.1	109,095	2.2	
Ownership	Proprietary	394,518	8.0	81,792	1.7	97,391	2.0	<0.0001
	Government	437,905	8.8	253,952	5.1	111,783	2.3	
	Non-profit	2,036,632	41.1	674,507	13.6	861,951	17.4	
Region	Northeast	977,438	19.7	393,630	8.0	359,458	7.3	<0.0001
	Midwest	472,866	9.6	187,381	3.8	293,557	5.9	
	South	977,254	19.7	332,773	6.7	163,643	3.3	
	West	441,497	8.9	96,467	2.0	254,467	5.1	
Season	winter	717,214	14.5	242,683	4.9	272,450	5.5	0.6944
	spring	754,419	15.2	274,944	5.6	280,908	5.7	
	summer	759,370	15.3	261,500	5.3	284,696	5.8	
	fall	638,052	12.9	231,124	4.7	233,071	4.6	
Length of stay	1–3 days	2,032,614	41.1	630,693	12.7	757,407	15.3	<0.0001
	4-6 days	469,322	9.5	198,943	4.1	178,016	3.6	
	7+ days	367,119	7.4	180,615	3.6	135,702	2.7	

* Percentages for each variable sum to 100% and are neither column nor row percents.

Results from fitting a Log-Normal model to the data are shown in Table 3. The unadjusted effect of race on the expected time of leaving AMA was about twice the adjusted effect. After controlling for the other covariates, the expected time to AMA discharge is 20% shorter for Blacks than whites. We selected the lognormal parametric survival model based on analysis of the Akaike Information Criterion AIC, which was minimized under the final adjusted model (AIC = 597,362.2). The unadjusted model that included only race had an AIC = 655,703.7. The variables which contributed to attenuating the effect of race on the expected time to AMA discharge included 18 to 44 age group and 45 to 64 age group (83% and 72% respectively), Self-pay patients, and Medicaid (75% and 64% respectively), mental illness (57%), males (50%), Northeast (44%), bedsize 100–199 (35% ), proprietary or government hospitals (21% and 20% respectively), and summer (15%). The year terms were included in the adjusted analysis but the estimates were not shown in Table 3. The most significant predictors included age, insurance coverage, mental illness, gender, and region. 

**Table 3:** Adjusted associations of patient and hospital characteristics with the expected time to AMA discharge.

Adjusted Associations with the Expected Time to AMA Discharge			
Variable	Categories	Expected Time to AMA Discharge	*p*-Value
Gender	Male *vs*. female	−50.01	<0.0001
Age	18–44 *vs*. Age 65+	−83.56	<0.0001
	45–64 *vs*. Age 65+	−72.25	<0.0001
Race	Blacks *vs*. Whites	−20.21	<0.0001
	Other *vs*. Whites	14.61	<0.0001
Insurance	Medicaid *vs*. Private Insurance	−64.58	<0.0001
	Medicare *vs*. Private Insurance	−40.51	<0.0001
	Self-Pay *vs*. Private Insurance	−75.89	<0.0001
	Other *vs*. Private Insurance	−48.27	<0.0001
Marital Status	Single *vs*. Married	−15.56	<0.0001
	Other *vs*. Married	−19.01	<0.0001
Diagnosis	Mental *vs*. non mental	−57.41	<0.0001
	Circulatory *vs*. non Circulatory	−9.57	<0.0001
	Digestive *vs*. non Digestive	4.59	0.0515
	Respiratory *vs*. non Respiratory	−14.59	<0.0001
	Poisoning *vs*. non Poisoning	17.76	<0.0001
	Endocrine *vs*. non Endocrine	−3.57	0.0597
	Infectious *vs*. non Infectious	8.92	0.0078
Region	Northeast *vs*. South	−43.48	<0.0001
	Midwest *vs*. South	−14.76	<0.0001
	West *vs*. South	−7.68	<0.0001
Bedsize	6–99 *vs*. 500+	−14.15	
	100–199 *vs*. 500+	−34.62	<0.0001
	200–299 *vs*. 500+	−25.26	<0.0001
	300–499 *vs*. 500+	−12.68	<0.0001
Ownership	Proprietary *vs*. Not-for-Profit	−21.15	<0.0001
	Government *vs*. Not-for-Profit	−19.60	<0.0001
Season	Fall *vs*. winter	−7.56	<0.0001
	Spring *vs*. winter	−10.53	<0.0001
	Summer *vs*. winter	−15.19	<0.0001

## 5. Discussion

To our knowledge, our study is the first to examine the relationship between race and the LOS for AMA patients using a large nationally representative dataset during 18-year time period (1988–2006). A key strength of this study is that a comprehensive picture of such a discharge has been considered by taking into account the sociodemographic, clinical, and hospital characteristics of such a discharge.

Our study shows a difference in LOS among the major racial groups of U.S. AMA populations. Our major finding that Black patients have significantly shorter lengths of stay suggests that they may not be getting the medical treatment necessary to fully treat their condition. This findings suggests the possibility of racial disparities where quality and delivery of medical care are affected. Racial effects are often equivocal, because race is a proxy for socioeconomic factors, financial factors, educational background, and insurance status [27]. Furthermore, our results that Black patients are leaving earlier than Whites after controlling for patient and hospital related variables suggests that they may be getting even less care and more likely at risk for readmission for the incomplete medical treatment and that their post AMA discharge outcomes may be even worse than whites. Our findings are consistent with previous studies who found that age, gender, and diagnosis have been identified as patient characteristics contributing to LOS [25,28]. Our results are in agreement with another study that found female gender is associated with longer LOS while younger age and marital status were associated with shorter LOS [29]. Our findings indicated that both sociodemographic and hospital characteristics contributed essentially to the prediction of LOS. The major predictors were age, gender, uninsured, Medicaid, Northeast, and mental diagnosis. These results identified the demographic, clinical, and hospital characteristics that moderated the relationship between race and length of stay for AMA patients. We found that patients with mental comorbidity leave the hospital faster than other patients. Previous study has shown that mental illness, in particular substance abuse, was associated with shorter length of stay which may be explained by patients being noncompliant with treatment or personality traits [30]. The results of the analyses contribute to the body of literature suggesting that certain demographic, clinical, and hospital characteristics may lead to shorter stays and thus increase the risk of AMA. LOS is not as simple and straightforward measure as it would appear to be, but is rather a multifaceted concept. If used inappropriately, LOS data would present information that is misleading [31].

A majority of interest of the AMA literature over the last three decades has been on psychiatric patients and especially those entered into substance abuse programs who left AMA. In particular, the 1980s’ peak in the number of studies appeared to coincide with the upsurge in patients' rights litigation in that decade [32]. Over the past two decades, the delivery of mental health services in the U.S. has undergone drastic changes due to the shift from institutional care towards community care, emergence of managed mental health care and increased use of psychotropic medications in the management of mental illnesses [33,34,35]. One major element of these changes was the reduction in the length of inpatient stays [34,36,37]. Inpatient care is the most expensive component of mental health services and accounts for most mental health expenditures [37]. Effective hospital utilization is therefore frequently conceptualized as the shortest length of stay necessary to manage symptoms and behaviors associated with mental illness [25]. What is worthy to note was that the shift to decreasing LOS following non-AMA admissions would generally be seen as a good starting point and an indicator of good quality of care where patients receive medical care for the optimum time period. However, an earlier AMA discharge among the AMA population could be seen as a marker of an inferior quality of care.

A previous study has shown that length and cost of hospital stay were significantly correlated [38]. It is important to examine factors that either facilitate or impede utilization of services among patients leading to AMA discharge because resource allocation, delivery of medical care, and quality of care are affected [3]. Preventing AMA discharge is likely to benefit both patients and health systems. Identifying these factors could assist in targeting interventions towards appropriate patients and potentially minimize AMA discharges, thus reducing the clinical, social, and economic impact on the healthcare system. The higher resource utilization during the follow-up period suggests that patients leaving under these circumstances may not have sufficiently recovered from their illness at the time of discharge because of incomplete treatment during the index admission [8]. LOS in hospital was also significantly longer during subsequent admissions for patients who left AMA [8]. LOS can reveal useful information about the performance of hospitals and it is not surprising that LOS has been accepted as a robust index of system performance [31]. Its value as a performance indicator is displayed in comparing service systems and following trends in utilization. LOS is an integral factor in the calculation of the hospital bed requirements for a given population [31]. In addition, its magnitude can be influenced by system as well as patient–related factors. 

There were several limitations in our study. It was retrospective in nature. We were unable to examine readmission rates for the patients since the data sources were based on discharges and not individuals. Despite these limitations, this study can be used as a point of departure for quality improvement efforts and should guide and improve prevention programs designed to prevent or reduce the burden of AMA. One of the effective ways to monitor hospital utilization is predicting LOS [28]. The results of this analysis may be in particular useful to health care providers and administrators in predicting such occurrences and will provide impetus for the development of intervention strategies. Furthermore, if the results of our study could lead to reduce the rate of AMA discharges, this would help in maximizing treatment resources. It is possible that other variables not examined in this study such as number of previous AMA discharges, socioeconomic status, education, employment, and type of facility (urban *vs.* rural hospital, teaching *vs.* community hospital) could have improved our understanding of the relationship between LOS and patient and hospital factors for AMA patients. In order to implement effective interventions, additional research is needed to more fully understand the mechanisms through which, predictors influence the association between length of stay and race among AMA patients. Our results highlight the need for longitudinal studies to identify the mechanisms behind leaving AMA. Future research could also evaluate whether intervention strategies to improve follow-up of patients who leave AMA provide any benefit in terms of outcomes or cost. 

## 6. Conclusions

Patient, clinical, and hospital characteristics explained partly the relationship between the length of stay and race for AMA patients. Understanding this relationship has policy implications for how health care is provided to AMA patients. These findings should be of interest to persons concerned with measuring and understanding health care utilization. Reduction in LOS raises questions about quality of inpatient care. Our results contribute to concerns that under-treatment may be concentrated in minority populations. Also, these findings may be beneficial for clinicians, mental health advocates, policy makers, researchers, and program planners interested in addressing the important issue of treatment-compliance problems. Future research would be to examine the effect of AMA discharges on delivery of services and their cost-effectiveness.
